# The burden of war-injury in the Palestinian health care sector in Gaza Strip

**DOI:** 10.1186/s12914-018-0165-3

**Published:** 2018-06-28

**Authors:** Marwan Mosleh, Koustuv Dalal, Yousef Aljeesh, Leif Svanström

**Affiliations:** 1 Department of Health Sciences, Mid Sweden University, Sundsvall, Sweden; 2 School of Health Sciences, Örebro University, Örebro, Sweden; 3 Higher School of Public Health, Al-Farabi Kazakh National University, Almaty, Kazakhstan; 4 Islamic University of Gaza, Gaza Strip, Palestine; 5 Professor (Emiratus), Karolinska Institutet, Stockholm, Sweden

**Keywords:** War, Injuries, War injuries, Disability, Palestine, Gaza Strip, Gaza war

## Abstract

**Background:**

War-related injury is a major public health concern, and a leading cause of mortality, morbidity, and disability globally, particularly in low and middle-income countries such as Palestine. Little is known about the burden of war-related injury in the Palestinian context. The objective of this study was to characterize the incidence and pattern of injuries, associated with war in Gaza Strip, from July 8 to August 26, 2014.

**Methods:**

This was a descriptive study based on an injury registry at hospital facilities in the Gaza Strip. A total of 420 victims records from 2014 Gaza war injuries were randomly selected, proportionate to the size of the study population estimated across five Gaza governorates. Simple descriptive statistics were calculated to explore the frequency and percentage distribution of study variables and injury data. A chi-square test (X^2^) was used. The significance level was derived at *p* < 0.05. The data were analyzed by IBM SPSS software, version 23.

**Results:**

Males (75.5%) have experienced more war-related injuries than females (24.5%), constituting a male: female ratio of 3.1:1. Almost half (49.5%) of the injured victims were of the age group 20–39, followed by children and adolescents (< 20 years), accounting for 31.4%. More than half of victims were single (53.6%), 44.3% were married and the rest were widowed or divorced. The overall number of injuries was 6.4 per 1000 population, though it varied among regions. North Gaza reported the highest number of injuries (9.0) and Rafah the lowest (4.7) per 1000 population. Blast and explosion were found to be the most common causes of war injuries (72.9%). The highest proportion of injuries were reported in the upper body. Multiple body shrapnel wounds and burns (39.3%) were most frequent. Other types of injuries were multiple organ injury (24.3%), fractures (13.6%), internal organ injury and bleeding (9.8%), amputation (4.5%), abrasions/lacerations and contusions (4.8%), vision or hearing loss or both (1.9%) and respiratory problems (1.9%). The highest percentage of injuries were classified as mild (46.9%), and the rest ranged from moderate-to-severe. Almost 26% of individuals had sustained disability, and most of them had physical/motor impairment.

**Conclusion:**

War-related injuries constitute a major problem to public health discipline and clinical medicine as well. A better surveillance system using ICD codes, and development of a comprehensive electronic data network are necessary to make future research easier and more timely.

## Background

Wars or armed conflicts and their impacts are a growing public health problem globally. They are a major cause of mortality, morbidity and disability, as well as a significant contributor to the disease burden, especially in low and middle income countries [1].War-related injuries place a substantial burden on persons, households and populations and increase poverty in communities [2]. Furthermore, the burden of injury is enormous for a community’s economy and health [3].

The World Health Organization (WHO) reported that about 15% of the burden of disease worldwide in 1990 was due to injuries [4]. A figure predicted to increase to 20% by 2020. The Global Burden of Disease report also predicted that, by 2010, 25% of resources in developing countries would be spent on healthcare and rehabilitation related to injuries [5, 6]. Injuries are also recognized as a common cause of disease burden in developed countries such as Europe and United States [4], as well as in Latin America and the Caribbean. The projections of mortality are expected to rise dramatically by 2030 [4]. Injuries resulting from different mechanisms of conflict or war kill more than 5 million annually and negatively impact the life of millions of people globally [1, 2]. These injuries are estimated to cause 9% of deaths worldwide and put demands on health resources in every country in the world [7]. There are thousands of hospitalizations related to injuries with different degrees of severity and disabilities [1, 7]. Many individuals who experience injuries due to acts of violence, conflicts, or other causes commonly sustain some kind of temporal or permanent disability; almost 16% of all disabilities reported globally; were due to injury [8].

The Palestinian territory, particularly Gaza Strip, has experienced frequent wars especially in the latest several years, resulting in thousands of injuries, deaths, and disabilities, as well as a large number of displaced people, and the destruction of health care facilities and infrastructure [9].

For instance, in the 2008 Gaza war, according to Ministry of Health (MOH) statistics and media outlets, an estimated 5300 Palestinians were injured, and 1419 deaths were documented by the Palestinian Centre for Human Rights during Israel’s military action in Gaza Strip during the same period. Among those injured, 30% were children and 16% were women [9]. In the 2012 Gaza war, 279 deaths and 2173 injuries related to war were reported by the Palestinian MOH [10, 11].

More recently, in the 2014 Gaza war, more than 2200 civilians were killed [12], and more than 11,000 were injured [13]. In addition, 17 hospitals and 50 primary health centers were damaged, and 6 hospitals and 28 primary health clinics had to close altogether; 16 ambulances were damaged, 83 health care staff were injured, and two health care staff were killed, all of which has created a large gap in the health care supply as well as a challenge to health care and disease management in Gaza health facilities [14, 15]. Indeed, the continuation of medical care and management for injured victims and patients with other disease has become a public health concern and poses a challenge to the Palestinian health system during and after emergency situations [16]. Injured persons who develop complications following initial treatment of injuries and discharge from hospital are also of public health concern in Gaza Strip [17]. There is insufficient national knowledge about this interesting health problem, as injury-related information is not always available and studies on the situation are lacking. Using a multi-hospital registry of injuries in Gaza, we characterize the incidence and pattern of war-related injuries in the Gaza Strip from July 8 to August26, 2014.

## Methods

### Study design

This was a descriptive study based on an injury registry maintained for Palestinian visitors to hospital facilities in Gaza Strip.

### Study population

The study population included all who sustained war-related injuries in Gaza’s five governorates during the 2014 Gaza war. All the medical records for war-related injuries were collected by the study sponsor, the Assalama Charitable Society for Wounded and people with Disability (ACSWD). Handwritten paper medical records included sex, marital status, place of residence, and mechanism of injury (blast/explosion, gunshot and other). We excluded non-violent or indirect war-related injuries, including road crashes, falls, domestic violence, work-related injuries, and the like. Any war-related injuries outside the 2014 Gaza war time period were also excluded.

### Sample size and sampling process

We randomly selected a representative sample of the registry’s 11,228 injuries for this study, owing to resource constraints. The selection of the study sample is based on proportional distribution of subjects in the five Gaza Strip governorates. We used the following formula to estimate the optimal sample size required for a study to assure adequate power to detect statistical significance [18].

$$ \mathrm{n}=\mathrm{N}/\left\{\left(\upalpha \right)2\right\}\mathrm{x}\left(\mathrm{N}+1\right) $$

where *α* = 0.05, N = total population and n = sample size selected for the study.

Applying the formula to the total of 11,228 injury records, we arrived at 400 cases, and the study sample was increased to *n* = 420 by survey and random selection of cases for inclusion. The study sample was selected randomly, proportional to study population size estimates across the Gaza governorates. The total sample was distributed across the five Gaza Strip governorates as follows: North Gaza: 117, Gaza: 141, Middle Gaza: 62, KhanYounis: 62 and Rafah: 38 cases.

### Data collection

The data were extracted from the handwritten injury/medical records, which included socio-demographic profiles of those injured in the 2014 Gaza war. The medical reports had not used any International Classification of Diseases (ICD) codes. The severity of injuries (mild, moderate, severe) was analyzed according to physician assessment in the report of injury form. Since, Physicians who treated and assessed the war-injury victims determined the severity of injury and other issues in the record based on their medical perceptions at the point of treatment. In this study, the first author led the data input process from patients records with the technical and administrative support of two other trained staff. The data input was independently checked and re-verified by a second person.

### Statistical analysis

Data were coded, entered, and processed using SPSS version 23. Simple descriptive statistics were used to explore the frequency and percentage distribution of study variables and injury data. A cross-tabulation was also used to compare the relationship between two variables. A chi-square test (X^2^) was used to derive *p*-values*. P < 0.05* was considered statistically significant, and 95% confidence intervals (CIs) of injury variables were also derived.

### Ethical aspects

We used available data without revealing any direct personal information about the injured victims. This study was approved by the Palestinian Health Research Council (approval No. PHRC/HC/61/15). Permission to use data was also obtained from the ACSWD and concerned authorities in Gaza Strip.

## Results

### Demographic data

In total, 420 study records were examined. War-related injuries were reported higher among males 75.5% (95% CI:70.6–79.3), than females 24.5% (95% CI:20.7–29.4), resulting in a male: female ratio of 3.1:1. The largest number of cases 49.5% (95% CI:44.8–54.3) were adults 20–39 years, 53.6% (95% CI:48.1–58.1) were single, 44.3% (95% CI:40.0–49.8) were married and the remaining were widowed or divorced (Table 1).

**Table 1:** Characteristics of war-injured persons (*n* = 420)

Characteristics		n	% (95% CI)
Sex	Male	317	75.5 (70.6–79.3)
	Female	103	24.5 (20.7–29.4)
Age group	0–19 years	132	31.4 (26.6–36.2)
	20–39 years	208	49.5 (44.8–54.3)
	40–59 years	65	15.5 (12.4–19.8)
	60+ years	15	3.6 (2.1–5.5)
Marital status	Single	225	53.6 (48.1–58.1)
	Married	186	44.3 (40.0–49.8)
	Widow	5	1.2 (0.2–2.4)
	Divorced	4	1.0 (0.2–2.0)
Governorate/Residency	North Gaza	120	28.6 (24.5–32.9)
	Gaza governorate	140	33.3 (28.9–37.7)
	Middle Gaza	62	14.8 (11.7–18.6)
	Khan Younis	61	14.5 (11.4–18.2)
	Rafah	37	8.8 (6.4–11.4)
Total		420	100.0

### War-related injury

The largest proportion of injuries (33.3%; 95% CI: 28.9–37.7) was reported from Gaza governorate, followed by North Gaza (28.6%; 95% CI: 24.5–32.9) (Table 1), which had the highest number of injuries (9.0 per 1000 population). Rafah governorate (the southern region of Gaza Strip) had the lowest reported number of injuries (4.7 per 1000 population) (Table 2), with an average number of injuries of 6.4 per 1000 population among all regions.

**Table 2:** Proportion and number of injuries per 1000 population (n = 420, *N* = 11,228)

Governorate/region	Estimated population^*^	Injured		
		Study subjects (n)	Estimated Injuries by population	Number of injury/1000 population
North Gaza	348,808	117 (27.9%)	3128	9.0
Gaza governorate	606,749	141 (33.6%)	3770	6.2
Middle Gaza	255,705	62 (14.8%)	1657	6.5
Khan Younis	331,017	62 (14.8%)	1657	5.0
Rafah	217,758	38 (9.0%)	1016	4.7
Total	1,760,037	420	11,228	6.4

Estimated population^*^ = According to the Palestinian Central Bureau of Statistics (2014 statistics)

**Fig. 1:**
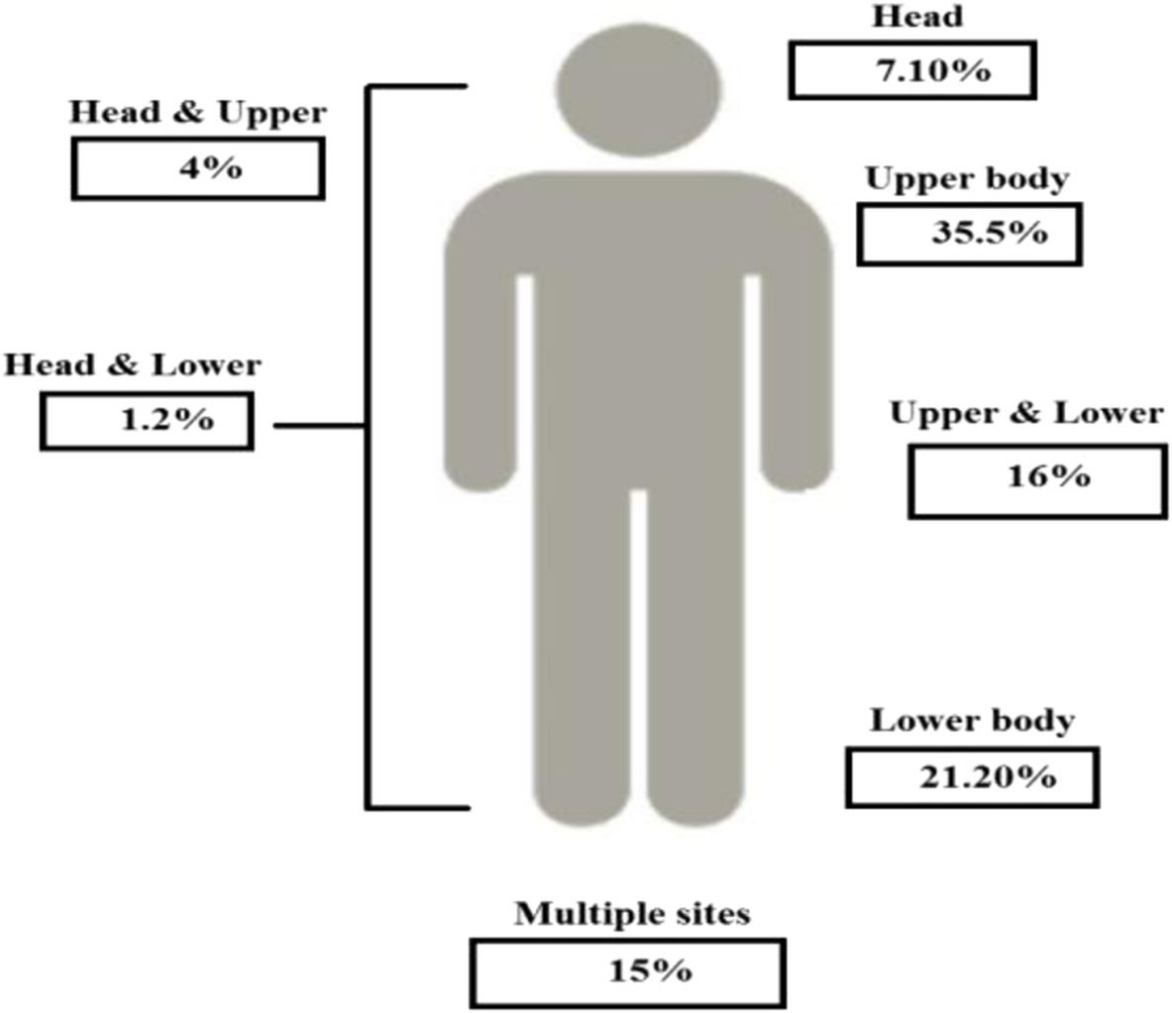
Anatomical site of war-related injury by type. Data from 2014 Gaza war

The highest percentage of injuries (35.5%; 95% CI: 31.0–40.1) were in the upper body, a figure that increases to 42.6% if head injuries (7.1%) are included (Table 3). This is followed by lower body (21.2%; 95% CI: 17.0–25.0), both upper and lower body (16.0%; 95% CI: 12.4–19.5), and multiple sites (15.0%; 95% CI: 11.9–18.3) (Table 3, Fig. 1).

**Table 3:** Injury characteristics of the study sample (*n* = 420)

Injury Information		*n*	% (95% CI)
Injury site	Upper body	149	35.5 (31.0–40.1)
	Lower body	89	21.2 (17.0–25.0)
	Upper and lower body	67	16.0 (12.4–19.5)
	Multiple sites	63	15.0 (11.9–18.3)
	Head	30	7.10 (4.9–9.8)
	Head and upper body	17	4.0 (2.4–6.3)
	Head and lower body	5	1.2 (0.2–2.4)
Mechanism/cause of injury	Explosion	306	72.9 (68.6–76.9)
	Gunshot	35	8.3 (6.0–11.5)
	Others	79	18.8 (15.5–22.5)
Nature of injury	Shrapnel, burns and wounds	165	39.3 (35.5–43.9)
	Multiple injuries	102	24.3 (20.2–28.4)
	Fractures	57	13.6 (10.2–16.7)
	Internal organ injury & bleeding	41	9.8 (6.2–13.8)
	Abrasion, lacerations, contusion	20	4.8 (2.6–6.7)
	Amputation	19	4.5 (2.6–6.9)
	Vision/hearing problems or both	8	1.9 (0.8–3.3)
	Respiratory problems	8	1.9 (0.7–3.3)
Severity of injury	Mild	197	46.9 (41.9–51.9)
	Moderate	109	26.0 (22.0–30.5)
	Severe	114	27.1 (23.6–31.2)
Information about war injury-related disability			
Disability	No disability	312	74.3 (70.2–78.8)
	With disability	108	25.7 (21.2–29.8)
Type of disability	Physical impairment	80	19.0 (15.0–22.7)
	Visual impairment	11	2.6 (1.2–4.3)
	Hearing impairment	12	2.9 (1.2–4.6)
	Multiple impairment	5	1.2 (0.2–2.4)
Total		420	100.0

The study found that blast or explosion was the most common mechanism of injury (72.9%; 95% CI: 68.6–76.9).

In terms of the nature of injury, war-related body shrapnel’, burns, and wounds comprised the highest proportion of injuries 39.3% (95% CI:35.5–43.9), followed by multiple injuries 24.3% (95% CI: 20.2–28.4), fractures 13.6% (95% CI: 10.2–16.7), internal organ injury and bleeding 9.8% (95% CI: 6.2–13.8), abrasions/lacerations and contusions 4.8% (95% CI: 2.6–6.7), amputations 4.5% (95% CI: 2.6–6.9), vision/hearing problems or both 1.9% (95% CI: 0.8–3.3), and respiratory problems 1.9% (95% CI: 0.7–3.3).

Most injuries were classified as mild 46.9 (95% CI: 41.9–51.9), and the rest ranged from moderate-to-severe (Table 3). Almost 26% (95% CI:21.2–29.8) of injured victims had sustained disability, and most had physical/motor impairment. Disability was classified as: physical impairment (19%), hearing impairment (2.9%), vision impairment(2.6%), and multiple impairments (1.2%) (Table 3).

### Injury management/procedure carried out for injured victims

Of the 420 injured victims surveyed during the study period, 49.5% (95% CI: 44.8–54.4) underwent debridement, shrapnel removal, and wound management, while 19.3% (95% CI:20.9–24.1) had surgery of the head, face, neck, chest, spine, pelvis, or extremities; 16.4% (95% CI:11.6–21.2) had fracture management; 5.0% (95% CI:2.9–7.1) had undergone urgent exploratory laparotomy; 3.6% (95% CI:1.9–5.7) received multiple interventions; 3.3% (95% CI:1.7–5.2) underwent skin grafting and burn management; and 2.9% (95% CI:1.2–4.5) received treatment for disease due to gas inhalation and various respiratory problems related to war (Table 4).

**Table 4:** Injury management (*n* = 420)

Injury Management information	*n*	% (95% CI)
Debridement and wound management	208	49.5 (44.8–54.4)
Surgery of the head, face, neck, chest, spine, pelvis or extremities	81	19.3 (20.9–24.1)
Fracture management	69	16.4 (11.6–21.2)
Urgent exploratory laparotomy	21	5.0 (2.9–7.1)
Multiple interventions	15	3.6 (1.9–5.7)
Burn management and grafting	14	3.3 (1.7–5.2)
Respiratory problem management	12	2.9 (1.2–4.5)
Total	420	100.0

### Severity of injury and disability according to age, sex, and residence

Severity of injury and disability differed significantly between male and female war-injured victims. There was also a significance difference in severity of injury and disability among war-injured victims according to their residency across the five Gaza governorates (Table 5).

**Table 5:** Severity and war-related disability according to age, sex, and residence

		Severity of injury				War-related disability		
		Mild	Moderate	Severe	Sig.	Without disability (no)	With disability (yes)	*P-value*
Age	*n*							
0–19 years	132	71 (36.0%)	28 (25.7%)	33 (28.9%)	.064	104 (33.3%)	28 (25.9%)	*.070*
20–39 years	208	85 (43.1%)	55 (50.5%)	68 (59.6%)		143 (45.8%)	65 (60.2%)	
40–59 years	65	33 (16.8%)	21 (19.3%)	11 (9.6%)		52 (16.7%)	13 (12.0%)	
60+ years	15	8 (4.1%)	5 (4.6%)	2 (1.85)		13 (4.2%)	2 (1.9%)	
Sex	*n*							
Female	103	60 (30.5%)	25 (22.9%)	18 (15.8%)	.014	88 (28.2%)	15 (13.9%)	*.002*
Male	317	137 (69.5%)	84 (77.1%)	96 (84.2%)		224 (71.8%)	93 (86.1%)	
Governorate	*n*							
North Gaza	120	67 (34.0%)	33 (30.3%)	20 (17.5%)	*.*000	101 (32.4%)	19 (17.6%)	*.009*
Gaza city	140	62 (31.5%)	27 (24.8%)	51 (44.7%)		99 (31.7%)	41 (38.0%)	
Middle Gaza	62	24 (12.2%)	16 (14.7%)	22 (19.3%)		41 (13.1%)	21 (19.4%)	
Khanyounis	61	29 (14.7%)	15 (13.8%)	17 (14.9%)		40 (12.8%)	21 (19.4%)	
Rafah	37	15 (7.6%)	18 (16.5%)	4 (3.5%)		31 (9.9%)	6 (5.6%)	

## Discussion

### Responding to challenge

We have examined war-related injuries in the Gaza Strip, which gives a perspective on the realities of Palestinian life. The study was conducted in response to the call for medical and epidemiological assessment of injuries during frequent wars in the region. The Palestinian health care system suffers from fragmentation, insufficient medical supplies, and limited resources, especially during emergency situations [19].

During the 2014 Gaza war, the Gaza Strip witnessed the highest rate of internal displacement (about 28% of the population) as a consequence of war [19, 20]. Most importantly, there were more than 11,000 injured victims reported, the concurrent destruction of medical facilities and health system infrastructure, and other barriers to healthcare access, making it difficult for most people in need of health care to access it [19, 21, 22].

The study found that injuries in the 2014 Gaza Strip war were 3-fold higher among males than females (male: female ratio of 3.1:1). The study findings support the figures of the official Palestinian MOH report on previous wars in Gaza Strip [22–24] and reports on injury during the Al Aqsa Intifada (uprising) in 2000–2006 [25]. In Palestine, generally the men are the breadwinners. When a male family member is injured or disabled, the whole family, including the children, suffers. Therefore, wars affect not only males but the whole family by depriving them of the means to obtain food.

Children under 20 years old were the second most affected group after young adults (20–39-year-olds) with respect to number of injuries. This group may in the near future become a challenge for the Palestinian health sector in terms of intervention, management, and rehabilitation.

The Gaza governorate reported a higher proportion of injuries than other governorates, supporting findings from previous studies that the region had the largest number of injuries during wartime in Gaza Strip [23]. However, considering the number of injuries per 1000 population, the highest was reported in North Gaza and the lowest in Rafah governorate (the southern region of Gaza Strip), indicating that North Gaza was hardest hit of the Gaza Strip regions. Other studies and MOH figures illustrate substantial variation in the number of injuries from one war/invasion to another and among regions in the same territory [9–15].

Our results are similar to those reported in the Palestinian territory [26] but inconsistent with those reported during conflicts in Libya and Kosovo, which showed an overall number of injuries lower than reported in the Gaza Strip but varying among regions [27, 28]. Our results also disagree with those reported in Iraqi conflicts, where injuries per 1000 population were higher than those reported in the Gaza Strip [29]. From our point of view, injury resulting from wars, invasions, or conflicts seems to have more negative consequences than the prolonged impact of such wars, especially as the incidence of injury increases over time in the Palestinian territories. The territory could plan to reorient its health care services to those areas with the higher injury incidence. Additionally, creating advanced field hospitals and adequately re-supplying them, especially during times of crisis and conflict, has the potential to save the lives of thousands of people.

The vast majority of injuries were due to blast or explosion, consistent with findings of the effect of conflict on injury patterns in Baghdad from 2003 [30].

The highest proportion of war-injury types were: war-related body shrapnel’, burns, and wounds, followed by multiple injuries, fractures, internal organ injury and bleeding, abrasions/lacerations and contusions, amputations, vision/hearing problems or both, and respiratory problems due to poisonous gas inhalation during wartime. The majority of injuries were classified as mild, the rest as moderate-to-severe. The figures show more severe injuries and disabilities than the corresponding figures reported during the 2008 and 2012 Gaza wars [9–11, 22, 31, 32], suggesting that the Palestinian health care system should focus on treating injury victims more efficiently along with people with other diseases. The number of injuries during the Gaza war of 2014 was nearly double that reported in the 2008 and 2012 Gaza wars. Therefore, the number of injuries during the 2014 Gaza war may have exceeded the emergency capacity of the Palestinian health care sector [9, 10].

We found that war-related physical injury was the most common cause of disability in the 2014 Gaza war, in line with a report from the WHO, revealing that physical injury is the leading cause of disability in the world [33]. Our study supports Damage Need Assessment reports that show a greater number of injuries and disabilities among children and women, many of them being left permanently disabled [34]. Moreover, these figures were also consistent with a study conducted in Kuwait that addressed injuries sustained during the Second Gulf War, which showed that the majority of severe injuries, particularly vascular injuries, were due to blasts or explosions [35]. The increasing severity of injuries in our study may have contributed significantly to the increasing proportion of disabilities among injured people, constituting a greater burden on the health care sector in Gaza. The severity of injury may considerably affect the patient’s physiological functions as well as quality of life, since the intensity of injuries could delay the recovery process and prolong the period of treatment, especially for those who had moderate-to-severe injury. It is also important to be aware that multiple bodily functions can be impacted by injuries. For example, persons with moderate-to-severe head injury typically experience problems in concentration, balance, and sustaining attention, as well as in cognitive skills and thinking [36]. They may be partially or permanently disabled. Therefore, national and international attention should be paid, and sufficient resources devoted and reoriented to reduce the potential burden of injuries on the Palestinian healthcare system. The Palestinians can learn and benefit from the experiences of other countries that have incurred high numbers of injuries during wars, such as Lebanon and Bosnia. Evidence from those countries demonstrates that the best approach to achieving better outcomes and a reduction in fatal injuries is to empower and enhance national practices, skills, and knowledge in how to deal with injury, as well as to make important resources available [37].

### Strengths and limitations

The primary limitation of this study is that, since health providers did not code injuries using ICD categories, there could be misclassification. Secondly, the data sources were hand-written/paper reports made during extreme war injury management under pressure and tension, which may produce inaccuracy and incompleteness. We believe that completing medical records on a computer is much easier than entering all details by hand in paper record-books. It should be noted that the MOH has recently collaborated with the Norwegian Institute of Public Health and the WHO to enhance and strengthen the Palestinian health system network in terms of reporting and registration. Unfortunately, the process has not been completed or even initiated due to the 2014 Gaza war, which has directly affected and restricted this significant effort, leading to delays in the implementation of the plan [38]. Another limitation of our study is that we were not able to derive the rate of injury due to absence of person-years. We instead computed number of injuries per 1000 population. The identification of the severity of injury was based on physicians perceptions as reported in records of injured patients. The diagnosis may differ from physician to physician without use of ICD codes for injuries, especially with the large number of injuries and limited time and resources in a very stressful situation. We have no further information on the persistence and lifetime severity of injury or disability, nor whether the victims have recovered. It is worth mentioning that in a lower and middle income country without an appropriate and computerized healthcare network, during emergencies such as wars, invasion, or conflicts, it is difficult for physicians to report ICD codes of injuries and provide necessary treatment to the war victims with poor medical facilities. Therefore the study has focused on available medical reports and highlighted the patterns of war injuries in the Palestinian healthcare sector in Gaza Strip, mainly to provide an overview of the issue. We recommend follow-up study of the victims to explore their actual disability and injury severity.

Despite the drawbacks, our study has addressed a significant health problem in the Palestinian healthcare sector, which has not been explored sufficiently in previous studies. Furthermore, as this study was implemented in the Gaza Strip governorates, it might be possible to generalize the findings to nearby regions and territories such as the West Bank, which are politically similar and identical in terms of their customs, ethnic backgrounds, and habits, and have been impacted by conflicts and wars, often at the same time period.

Although this study has provided important findings on the Palestinian healthcare sector, it has failed to suggest any health systems-related solutions for better treating war injury victims in the Palestinian health system.

### Implications of the study findings

Our findings may be of direct significance to Palestinian health care by providing useful information on the provision of health care and medical services for injured persons. It is possible that, by giving an overall picture of the patterns of injuries, we may help to facilitate injury management and treatment in Palestinian health care facilities. The healthcare plan should include an immediate, appropriate, and effective intervention and should be incorporated in the general practice to reduce the negative impact of large numbers of injuries on the quality of health care in the Palestinian health sector.

Most importantly, this study could serve as the baseline for further research on Palestinian health care, especially in Gaza Strip. There is little information available on war-related injuries. The current study has focused on incidence and patterns of injuries, causes/mechanisms of injury, disability, and management of injuries. The research community has received vital information on war injuries from the current study.

A new effective strategy should be adopted that would include health education and improving awareness of the possible risks of injury and the necessity of providing suitable assistance and care at the moment of injury, so that the fatality rate of injuries can be reduced. An important strategy is awareness concerning, and avoidance of, hazardous materials during and after wars, which could significantly reduce injury. To make a prevention strategy as successful as possible, the media, as well as the community must engage in the process via special programs and campaigns. Finally, as the war injuries and disabilities addressed in this study have been shown to constitute a great burden, they must be placed among the priorities of national and international health policy. It is hoped that the study’s findings will create a deeper understanding of the burden and characteristics of this significant health problem and contribute to health planning and development processes.

## Conclusion

The study findings revealed that war-related injuries are a significant burden on Palestinian health facilities. The incidence of injury in modern war is 3 times that reported in previous wars and varies according to region and mechanism of injury. Our study suggests that better surveillance systems using ICD codes, and development of a comprehensive electronic data network are necessary to make future research easier and more timely.
