# Correction to: The burden of war-injury in the Palestinian health care sector in Gaza Strip

**DOI:** 10.1186/s12914-018-0171-5

**Published:** 2018-08-13

**Authors:** Marwan Mosleh, Koustuv Dalal, Yousef Aljeesh, Leif Svanström

**Affiliations:** 1 Department of Health Sciences, Mid Sweden University, Sundsvall, Sweden; 2 School of Health Sciences, Örebro University, Örebro, Sweden; 3 Higher School of Public Health, Al-Farabi Kazakh National University, Almaty, Kazakhstan; 4 Islamic University of Gaza, Gaza Strip, Palestine; 5 Professor (Emiratus), Karolinska Institutet, Stockholm, Sweden

## Correction

In the original publication of this article [1] the background section contained an inaccurate mention of the amount of civilian deaths in the 2014 Gaza war. In this correction article the incorrect and correct information are published for clarification.

Incorrect:

- More recently, in the 2014 Gaza war, more than 2200 civilians were killed [12]

Corrected:

- More recently, in the 2014 Gaza War, according to a report from the Palestinian Health Information Center [12], there were 2200 deaths. The majority were civilians

Further information related to the report from Palestinian Health Information Center can be accessed from the original publication.
